# Serum glucose, lactate dehydrogenase and hypertension are mediators of the effect of body mass index on severity of COVID‐19

**DOI:** 10.1002/edm2.215

**Published:** 2021-02-16

**Authors:** Huadong Yan, Amrita Vijay, Fanrong Jiang, Nanhong Zheng, Yaoren Hu, Honghua Ye, Benjamin Ollivere, Ting Cai, Ana M Valdes, Guruprasad P. Aithal

**Affiliations:** ^1^ Department of Infectious Diseases Key Laboratory of Diagnosis and Treatment of Digestive System Tumors of Zhejiang Province Hwamei Hospital University of Chinese Academy of Sciences Ningbo China; ^2^ Ningbo Institute of Life and Health Industry University of Chinese Academy of Sciences Ningbo China; ^3^ NIHR Nottingham Biomedical Research Centre Nottingham University Hospitals NHS Trust and University of Nottingham Nottingham UK; ^4^ Division of Rheumatology, Orthopaedics and Dermatology School of Medicine University of Nottingham Nottingham UK; ^5^ Department of Pharmacology Hwamei Hospital University of Chinese Academy of Sciences Ningbo China; ^6^ Department of Cardiology Hwamei Hospital University of Chinese Academy of Sciences Ningbo China; ^7^ Department of Experimental Medical Science Key Laboratory of Diagnosis and Treatment of Digestive System Tumors of Zhejiang Province Hwamei Hospital University of Chinese Academy of Sciences Ningbo China; ^8^ Nottingham Digestive Diseases Centre School of Medicine University of Nottingham Nottingham UK

**Keywords:** biochemical markers, BMI, COVID‐19, disease severity

## Abstract

**Background:**

COVID‐19 has a broad clinical spectrum. We investigated the role of serum markers measured on admission on severity as assessed at discharge and investigated those which relate to the effect of BMI on severity.

**Methods:**

Clinical and laboratory data from 610 COVID‐19 cases hospitalized in the province of Zheijang, China were investigated as risk factors for severe COVID‐19 (assessed by respiratory distress) compared to mild or common forms using logistic regression methods. Biochemical markers were correlated with severity using spearman correlations, and a ROC analysis was used to determine the individual contribution of each of the biochemical markers on severity. We carried out formal mediation analyses to investigate the extent of the effect of body mass index (BMI) on COVID‐19 severity mediated by hypertension, glycemia, Lactose Dehydrogenase (LDH) at the time of hospitalization and C‐Reactive Protein levels (CRP), in units of standard deviations.

**Results:**

The individual markers measured on admission contributing most strongly to prediction of COVID‐19 severity as assessed at discharge were LDH, CRP and glucose. The proportion of the effect of BMI on severity of COVID‐19 mediated by CRP, glycemia or hypertension, we find that glucose mediated 79% (p < .0001), LDH mediated 78% (p < .0001), hypertension mediated 66% (p < .0001); however, only 44% (p < .005) was mediated by systemic inflammation (CRP).

**Conclusion:**

Our data indicate that a larger proportion of the effect of BMI on severity of COVID‐19 is mediated by glycemia and LDH levels whereas less than half of it is mediated by systemic inflammation.

## Graphical abstract

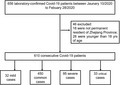

## 1 INTRODUCTION

The clinical spectrum of SARS‐CoV‐2 infection appears to be wide, encompassing asymptomatic infection, mild upper respiratory tract illness with cough, fever and fatigue to more severe viral pneumonia with a risk of consequent respiratory failure and death. Risk factors associated with symptomatic disease requiring hospitalization are uncertain and factors that influence the risk of developing severe disease remain incompletely understood. Age has been consistently associated with increased mortality^1^; type 2 diabetes and hypertension, two most prevalent comorbidities in hospitalized patients have been also associated with severe manifestations.^2^, ^3^, ^4^

Recent data coming from the US and Western Europe show that being overweight or obese is a significant risk factor associated with hospitalization,^5^ requirement to mechanical ventilation^6^, ^7^ and in‐hospital mortality from COVID‐19.^1^ A number of mechanisms may underlie association of body mass index (BMI) and pneumonia including increased airway resistance and reduced respiratory muscle strength, lung volumes, and gas exchange.^8^ However, it has emerged that patients with high BMI are at greater risk of death from COVID‐19 than patients with asthma.^1^ This suggests that the mechanisms by which SARS‐Cov2 develops into a severe form of COVID‐19 may be directly related to metabolic pathways that are specifically dysregulated in obese or overweight individuals. Obesity is also a key risk factor for type 2 diabetes, hypertension and cardiovascular disease all of which have been associated with COVID‐19. Furthermore, metabolic impairment characterized by insulin resistance, hyperglycaemia and dyslipidaemia may remain unrecognized in substantial proportion of the population.^9^ Evaluating of these parameters is important to risk stratify patients with COVID‐19.^10^

The objective of this study was to identify risk factors for symptomatic COVID‐19 and the development of its severe/critical form using a multicentre retrospective cohort of 610 patients with COVID‐19 from Zhejiang province, China. In particular, we have investigated to what extent the effects of high BMI (BMI of ≥ 23 kg/m^2^).^11^ of COVID‐19 severity in the current cohort are mediated by systemic inflammation and how much by LDH, glycemia or hypertension.

## 2 PATIENTS AND METHODS

The study protocol conformed to the ethical guidelines of the 1975 Declaration of Helsinki. The local ethics committees of all hospitals approved the retrospective study of cohorts COVID‐19. The requirement for written consent was waived due to the retrospective and anonymous nature of this study.

### 2.1 Cohort of cases

**FIGURE 1:**
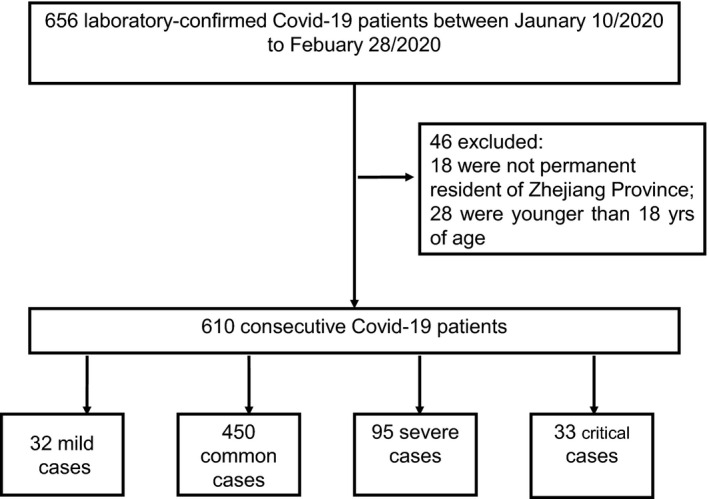
Flow chart of recruitment path for research study participants

Consecutive patients aged 18 years and over presenting to 14 hospitals in Zhejiang province, China between Jan 10 and Feb 28, 2020 and confirmed diagnosis of COVID‐19 infection were included (Figure 1). The diagnosis of COVID‐19 was made in accordance with the Guidelines for the Diagnosis and Treatment of New Coronavirus Pneumonia (fifth edition) formulated by the National Health Commission of the People's Republic of China.^12^ Confirmed case of COVID‐19 was defined as a positive result on real‐time reverse transcriptase‐polymerase chain reaction (RT‐PCR) assay of nasal and pharyngeal swab specimens. Only laboratory‐confirmed cases were included in the study. The clinical data of all patients were collected from the electronic medical records. All patients were administered with antiviral and supportive treatments, and prevention of complications based on their clinical conditions.

### 2.2 Clinical categories

The severity of the disease was classified according to the Guidelines for the Diagnosis and Treatment of New Coronavirus Pneumonia (fifth edition)^12^ and categorized into 4 groups as described previously.^13^ In brief, patients were classified into the following groups:

1. Mild: patients with mild clinical symptoms and no pulmonary changes on CT imaging
2. Common: patients with symptoms of fever and signs of respiratory infection, and having pneumonia changes on CT imaging
3. Severe: patients presenting with any one item of the following: a) respiratory distress, respiratory rate ≥ 30/min, b) oxygen saturation of finger ≤ 93% in resting condition, and c) arterial partial pressure of oxygen (PaO2) /oxygen concentration (FiO2) ≤ 300 mmHg (1 mmHg = 0.133 kPa)
4. Critical: patients meeting any one of the following criteria: a) respiratory failure requiring mechanical ventilation, b) shock and c) ICU admission requirement due to multiple organ failure

### 2.3 Analyses of severity

Individual demographic and clinical features were tested for association with the 4‐point scale of severity described above using a Spearman's correlation. Those variables that were significantly associated with the categorical measure of severity were then included in a LASSO logistic regression to select specific features to test as predictors of severe outcome (grade 3 or 4 of the categorical scale) vs less severe outcome (grade 1 or 2).

### 2.4 Laboratory methods

Clinical laboratory test results, including SARS‐CoV‐2 RNA detection results, biochemical indices, blood routine results, were collected from routine clinical practice across all 14 centres. SARS‐CoV‐2 RNA was detected using real‐time quantitative PCR (qPCR) on nucleic acid extracted from upper respiratory swab samples. Upper respiratory swab samples were collected on all suspected cases of SARS‐CoV‐2 infection on admission and immediately placed into sterile tubes with viral transport medium. All biochemical and haematology parameters were obtained via standard automated laboratory methods and using commercially available kits following to the manufacturer's protocols. Clinical and laboratory data are those collected and recorded at the time of admission.

### 2.5 Statistical methods

Categorical variables were expressed as frequency and percentages. Continuous variables were expressed as mean and standard deviations. Logistic regressions were carried out either unadjusted or adjusting for covariates as indicated in the table legends. p < .05 was considered statistically significant. To formally assess the contribution of CRP, LDH, glycemia and hypertension to the association between BMI and disease severity, we conducted mediation analysis using the ‘mediation’ package in R version 3.6.1 where we considered the various clinical traits as mediator for the causal effect of BMI on severity.

Analyses were carried out using R version 3.6.1. p < .05 was considered statistically significant.

## 3 RESULTS

The selection of the study population was carried based on a previously published protocol.^13^

We proceeded to investigate the biochemical and clinical factors that correlate with the disease severity. The demographic, clinical presentation features and biochemical markers in each of the four severity categories are shown in Table 1. We found that BMI greater than 23 kg/m^2^ was strongly associated with a more severe form of the disease, but BMI below 23 kg/m^2^ was not. We also found significant associations with male sex and older age. In terms of the biochemical measures, we found glucose and LDH having the most dramatic effect on severity compared to others. The presence of hypertension was significantly associated with severity, but type 2 diabetes was not.

**TABLE 1:** Association between demographics, biochemical measures, comorbidities and severity. odds ratios were derived by logistic regression severe (severe + critical) vs not severe (mild + common). p‐values adjusted for age, sex and BMI, except those for age, sex and BMI

Severity category	1 (mild)	2 (common)	3 (severe)	4 (critical)	adj OR severe vs not	95% CI		p‐value
Demographic & clinical parameters								
n	32	450	95	33				
Sex M	19 (59.3%)	207 (46.0%)	61 (64.2%)	25 (75.7%)	2.3	1.5	3.5	**<.0001**
Age years mean (SD)	40.8(12.4)	47.2(13.5)	53.7 (13.4)	62.4 (14.7)	1.0	1.0	1.0	**<.0001**
BMI kg/m^2^ mean (SD)	23.3 (3.5)	23.6 (3.3)	25.5 (3.6)	25.0 (3.5)	1.1	1.1	1.2	**<.0001**
BMI < 23 kg/m^2^ (SD)	20.2(1.2)	21.8 (1.5)	20.4 (2.0)	21.3 (1.5)	0.1	0.7	1.3	.955
BMI > 23 kg/m^2^ (SD)	25.7(2.6)	25.8(2.5)	26.6 (2.8)	26.4 (2.9)	1.1	1.0	1.2	**.001**
Smoker (%)	14 (43.7%)	61 (13.7%)	26 (27.3%)	14 (42.4%)	0.7	0.5	0.9	.076
Cough (Respiratory Symptoms) (%)	18 (56.2%)	27 (6%)	75 (78.9%)	30 (91%)	0.9	0.6	0.1	**.001**
Digestive Symptoms (%)	7 (21.8%)	52 (11.5%)	17 (17.9%)	4 (12.1%)	0.8	0.6	0.9	.089
Fever (%)	23 (71.8%)	366 (81.5%)	84 (88.4%)	28 (84.8%)	0.8	0.6	0.1	.573
Peak Temperature (SD)	37.5 (0.6)	37.8 (0.7)	38.0 (0.8)	38.2 (0.9)	1.1	1.0	1.1	**.006**
Biochemical measures								
Glucose mmol/l (SD)	6.0 (1.8)	6.2 (2.1)	6.8 (2.8)	9.0 (3.9)	1.3	1.1	1.4	**<.0001**
CRP mg/L (SD)	4.3 (12.6)	15.8(20.0)	22.3 (24.7)	48.8 (34.2)	1.0	1.0	1.0	**<.0001**
Lymphocyte (10^9^/L) (SD)	1.4 (0.6)	1.2(0.5)	1.1 (0.5)	0.7 (0.4)	0.1	0.08	0.2	**<.0001**
Monocyte (10^9^/L) (SD)	0.5 (0.3)	0.4 (0.2)	0.4 (0.2)	0.4 (0.3)	0.7	0.3	1.6	.519
Neutrophil (10^9^/L) (SD)	3.6 (1.5)	3.2 (1.5)	4.9 (5.5)	6.5 (3.4)	1.2	1.2	1.4	**<.0001**
WBC (10^9^/L) (SD)	5.6 (2.0)	5.0 (2.1)	6.2 (5.2)	7.0 (3.3)	1.2	1.1	1.3	**<.0001**
PLT (10^9^/L) (SD)	208.2 (54.2)	198.2 (70.7)	191.4 (63.9)	172.8 (84.5)	0.9	0.9	1.00	.079
HBG* (g/L) (SD)	142.3 (11.8)	135.0 (15.4)	136.0 (17.8)	131.8 (19.5)	0.9	0.9	1.01	.724
AST (iu/L) (SD)	21.6 (10.2)	26.9 (16.1)	36.8 (21.4)	47.7 (33.7)	1.03	1.02	1.04	**<.0001**
ALT (iu/L) (SD)	27.6(23.3)	27.5 (24.5)	38.6 (33.5)	39.5 (36.6)	1.01	1.0	1.02	**.0001**
ALB (g/dl) (SD)	42.0 (4.2)	41.4 (5.3)	36.1 (4.8)	31.1 (3.7)	0.7	0.6	0.8	**<.0001**
TC (mmol/L) (SD)	4.6 (0.9)	3.9 (0.8)	3.9 (0.8)	3.8 (1.3)	0.9	0.6	1.2	.600
TG (mmol/L) (SD)	2.5 (3.1)	1.4(0.7)	1.3 (0.4)	1.3 (0.5)	0.8	0.5	1.2	.481
HDL (mmol/L) (SD)	1.0(0.2)	1.1 (0.3)	1.0 (0.2)	1.0 (0.3)	0.3	0.1	0.8	**.026**
LDL (mmol/L) (SD)	2.7 (0.7)	2.2 (0.7)	2.1 (0.6)	2.0 (1.0)	0.7	0.5	1.1	.177
Potassium (mmol/L) (SD)	3.8 (0.3)	3.9 (2.4)	3.8(0.5)	3.6 (0.4)	0.7	0.4	1.0	.242
Sodium (mmol/L) (SD)	138.9 (2.1)	138.3 (2.8)	136.9 (3.2)	135.5 (7.5)	0.8	0.7	0.9	**<.0001**
Creatinine kinase (iu/L) (SD)	69.9 (10.5)	67.6 (18.7)	83.7 (52.3)	95.9 (230.)	1.01	1.0	0.02	**.014**
LDH (iu/L) (SD)	164.4 (41.2)	216.5 (72.7)	329.9 (146)	382.2 (166.5)	1.01	1.01	1.02	**<.0001**
Comorbidities								
Type 2 Diabetes	2 (6.2%)	37 (8.2%)	16 (16.8%)	5 (15.1%)	1.2	0.6	2.2	.565
Hypertension	3 (9.3%)	77 (17.1%)	38 (40.0%)	19 (57.5%)	2.3	1.4	3.7	**.0004**

* HBG 1 g/L = 0.1 g/dl. Bold values represent statistical significance

**FIGURE 2:**
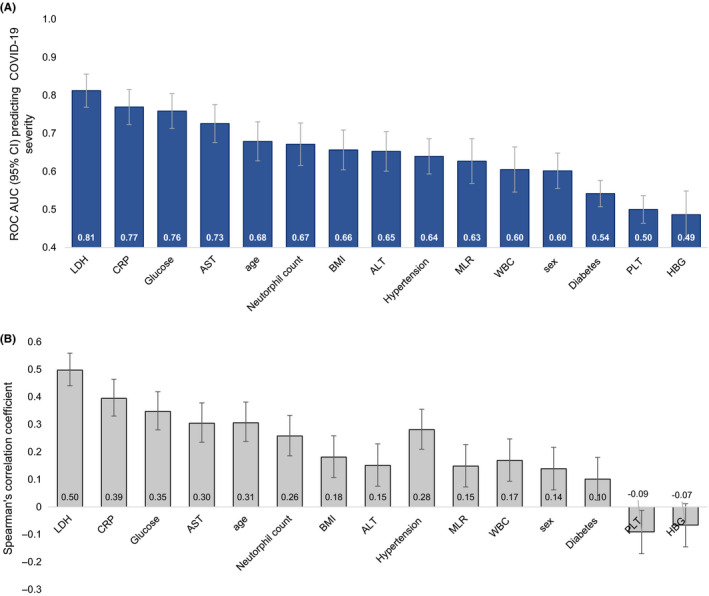
(A) ROC AUC (95% CI) and (B) Spearman correlations for biochemical and demographic characteristics ranked in order of predicting COVID‐19 severity

We further ranked the biochemical markers correlated with severity and put them side by side with some of the demographic characteristics in terms of how much they can explain severity on a ROC analysis including each variable independently (Figure 2). We found that levels of LDH, CRP and glucose on admission were each individually able to predict 75% or more of COVID‐19 severity. Furthermore, we found that LDH was a greater predictor of severity (beta 0.426; 0.320–1.870 95% CI; p < .001) compared to glucose (beta 0.264; 0.188–1.495 95% CI; p < .001) and CRP alone 0.20; 0.106–0.238 95% CI; p < .001).

Given the strong links between CRP, glucose and LDH with BMI, we decided to investigate whether the relationship observed in other cohorts between BMI and COVID‐19 severity might actually be mediated by these factors.

We next hypothesized that part of the association of BMI with severity is mediated by high glucose levels (hyperglycaemia), CRP and LDH. In order to test the mediatory effect of these variables, we carried out a formal mediation analysis where LDH, hyperglycaemia (glucose > 8mmol/l) and CRP were fitted as mediators of the effect of BMI on severity. We observed a partial mediation effect for glucose and CRP where both the average causal mediation effect (ACME) and average direct effect (ADE) were significant (Table 2). However, glucose mediated 79% (p < .0001) and CRP mediated 44% (p < .005) of the effect of BMI on severity (Table 2). LDH mediated 78% (p < .0001) of the effect of BMI on severity. For comparison, we also explored the mediatory effect of hypertension, given its strong links to metabolic syndrome and found that it mediated 66% (p < .001) of the effect of BMI on severity as shown in Table 2. We also carried a regression analysis of severity with glucose and BMI and found significant associations for glucose (beta = 0.25, 0.89–1.53 95% CI, p=<0.001) and for BMI (beta = 0.14, 0.33–1.25 95%CI, p = .001). Furthermore, a regression of glucose on BMI was also found to be significant (beta = 0.14, 0.77–0.27 95% CI, p = .001).

**TABLE 2:** Summary statistics of mediation analysis for the various clinical traits

Clinical trait	Effect	ACME	95% CI	p	ADE	95% CI	p	%
Hyperglycaemia	Partial	0.2105	0.085–0.26	<.0001	0.0484	0.02–0.07	<0001	79%
CRP	Partial	0.0412	0.011–0.17	<.05	0.1047	0.06–0.15	<.001	44%
LDH	Full	0.151	0.09–0.21	<.0001	0.042	−0.03	.13	78%
Hypertension	Partial	0.1863	0.083–0.23	<.0001	0.0632	0.03–0.10	<.001	66%
Hyperglycaemia + Hypertension	Partial	0.3954	0.22–0.57	<.0001	0.0947	0.06–0.14	<.05	86%
Hyperglycaemia + CRP	Partial	0.2515	0.11–0.27	<.0001	0.0917	0.01‐0.15	<.05	81%

Note

Models were built in to assess the effect of association between overweight/obese (BMI > 23) and severity mediated by clinical traits. The effect observed is described in the ‘effect’ column where: Null, corresponds to no association observed between high BMI ad severity mediated by the traits of interest. Partial, the association between the high BMI and severity is partially mediated by the clinical trait of interest. Direct, the association between high BMI and severity is fully mediated by the clinical trait of interest.

**FIGURE 3:**
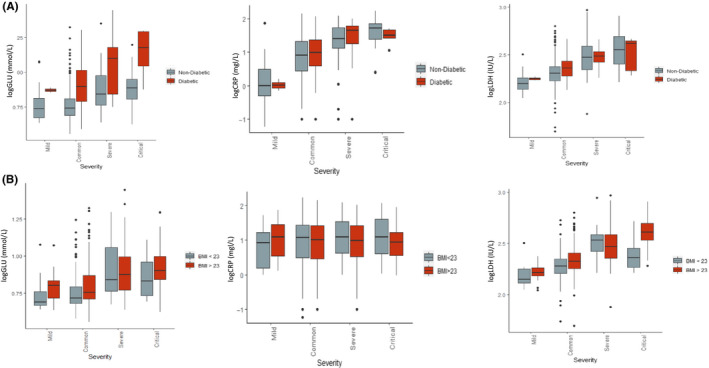
Glucose, CRP and LDH levels and its association with severity in (A) individuals with and without type 2 diabetes in COVID positive cases; (B) individuals in the overweight/ obese category (BMI > 23) compared to normal weight (BMI < 23) in COVID positive cases. Values for glucose, CRP and LDH represent log transformed values

Finally, although diabetes was not a strong risk factor for severity, it could be that the high levels of glucose seen to be predictive of severity may be only seen in diabetic patients. Therefore, we further investigated its effect in patients with and without type 2 diabetes and in the overweight/obese (BMI > 23 kg/m^2^) and normal weight (BMI < 23 kg/m^2^) categories. We found that glucose was significantly associated with severity in patients with and without type 2 diabetes (Figure 3). Similarly, the association of glucose with severity was significant in the overweight/obese category compared to the normal weight category. Furthermore, we found that CRP, a common marker of systemic inflammation, was significantly associated with severity in the presence or absence of type 2 diabetes and amongst normal and overweight categories as shown in Figure 3. A similar pattern was seen with regards to LDH levels.

## 4 DISCUSSION

Using a multicentre cohort of 610 patients from Zhejiang province, China we have identified key baseline features from demography (BMI and male sex), comorbidities (hypertension), 12, blood counts (neutrophil counts) and serum biochemistry (glucose, CRP, LDH) that were associated with severity of COVID‐19.

Recently, obesity (BMI of > 30 kg/m^2^) has been associated with increased risk of admission to acute and critical care settings^5^, ^6^, ^7^ as well as in‐hospital mortality^1^ in a cohort of patients from a hospital in New York and UK. In the current cohort (n = 610), mean (SD) BMI is distinctly lower at 23.98 (3.47) kg/m^2^ and ranged from 23.35 (3.50) kg/m^2^in those who had mild form to 25.02 (3.50) kg/m^2^ in the group who developed critical form of COVID‐19. This indicates that the relationship of rising BMI in this cohort is unlikely to be due to reduced cardiorespiratory reserve associated with obesity. It is important to note that Asians have a higher percentage of body fat than white people of the same age, sex and BMI, hence, are deemed to be at increased risk of its metabolic consequences at a BMI of ≥ 23 kg/m^2^.^11^ When we grouped people using a BMI cut‐off of 23 kg/m^2^, those with a BMI ≥ 23 kg/m^2^; mean (SD) had a higher risk to developing more severe form of the disease which increases from 25.77(2.62) in mild to 25.81(2.53) in common, 26.60 (2.88) in severe and 26.40 (2.98) in critical forms of the disease. On the other hand, there was no association between BMI and severity in those with a BMI < 23 kg/m^2^.

We have carried out formal mediation analyses to investigate the extent to which the effect of body mass index (BMI) on COVID‐19 severity is mediated by glycaemia, LDH, CRP and hypertension. Both serum glucose and CRP have been functionally linked to higher BMI in the general population.^14^, ^15^, ^16^ Obesity‐induced meta‐inflammation is associated with systemic increases in circulating inflammatory cytokines and acute phase proteins such as CRP and recruitment of leucocytes to inflamed tissues.^17^ Obesity has also been shown to impair immune response to influenza virus.^18^ When we investigated the proportion of the effect of BMI mediated by these two factors, we found that a higher proportion of the effect of BMI on COVID‐19 severity is mediated by glycaemia than by CRP. Our mediation analysis also finds that the effect of BMI is also mediated by hypertension more so than by CRP (Figure 3). Previous meta‐regression analysis has shown in African American and white individuals under the age of 50 with a median BMI of 29 found that hypertension and diabetes did not affect the relationship between obesity and severity.^19^ However, in our study population, half of the patients were 50 or older with a median BMI of 23.9 and only 4 individuals in our patient cohort had a BMI > 35. It is therefore possible that in younger morbidly obese individuals, the effect of morbid obesity may not be mediated by hypertension or diabetes. It is also possible that these effects may be related to ethnicity. Increased blood glucose levels at a cellular level can result in mitochondrial injury and endothelial dysfunction by generating reactive oxygen species and inhibiting nitric oxide production, respectively.^20^ Hence, glucose in conjunction with hypertension mediates the effect of BMI on COVID‐19 severity.

Serum LDH and glucose levels at the time of hospital admission are associated with severe/ critical form of COVID‐19 in our cohort and mediate the majority of the effect of BMI on severity; higher glucose levels are a risk factor for severe form of the disease irrespective of pre‐existent diagnosis of diabetes and across the full range of BMI. Therefore, it is plausible that glucose levels rise in response to stress related to the SARS‐CoV‐2 infection. A small cohort of 166 COVID‐19 patients from a single centre has shown fasting plasma glucose to be associated with severe form and longer hospital stay for those with and without diabetes.^21^ Acute hyperglycaemia can in turn significantly alter innate immune responses to infection, and this potentially explains some of the poor outcomes in hospitalized patients who develop hyperglycaemia as a consequence of bacterial and viral infections^22^ The role of LDH is likely to fit within the same pathway, as increased levels of LDH‐A are seen in immunological Warburg effects when immune cells carry out glycolysis even in the presence of oxygen, resulting in a strong pro‐inflammatory phenotype.^23^

Risk stratification of patients at the time of diagnosis would assist planning of interventions for patients at risk of developing severe disease. Important role of baseline glucose levels in determining the development of severe COVID‐19 indicates the potential benefits of prioritizing glycaemic control at the time of diagnosis and during hospitalization.^24^ Our results have important public health implications as they suggest that promoting interventions targeting glycaemia^25^ may prevent some of the worst COVID‐19 outcomes seen among overweight and obese individuals,^26^, ^27^ in particular those with prediabetes^28^ and ethnic minority communities^29^ where metabolic consequences of BMI are distinctly higher.

Limitations of our study include the retrospective nature of the COVID‐19 cohort, which inevitably lead to possible variations between hospitals involved and under‐recording of some of the symptoms. However, we have included consecutive cases from each of the secondary care hospitals to reflect the full range of case‐mix seen in routine clinical practice. Case definition and assessment of severity of COVID‐19 were adherent to national guidelines consistently across all the centres involved in this study.^11^ Although 128 of 610 patients included developed severe or critical form of the disease, mortality rate of < 1% means that we are unable to model prognostic factors related to most severe outcomes related to COVID‐19. In addition to the above, BMI using Asian overweight cut‐off points may limit generalizability to the wider population. The current study addressed the biochemical factors affecting outcome that might mediate the effect of obesity. We have chosen hypertension and diabetes as two of the possible mediators showing that glycemia and LDH levels are much stronger predictors. In addition, no information on IL‐1, IL‐6 or other inflammatory markers was available to test the role of BMI via other inflammatory markers. Information on other comorbidities such as COPD and chronic kidney disease was unavailable and therefore not accounted for in the analysis. It may be that other comorbidities have a stronger mediating effect on COVID‐19 severity but based on data from OpenSafely^1^ this appears unlikely. A further limitation to our study is that although biochemical/haematological methods may not have necessarily been identical at all 14 sites and this may have introduced some error. This would tend however to reduce the power to detect significant associations and is unlikely to bias our results as there were not any sites with significantly larger numbers of severe cases.

In conclusion, we find that baseline patient‐related and laboratory parameters in combination can be used to risk stratify COVID‐19 patients. Glycaemia and LDH levels have key roles in mediating the effect of BMI on the severity and outcome of SARS‐CoV‐2 infection and should be considered a target for public health measures to reduce the impact of the pandemic. The current findings have important clinical implications as they suggest that the cardiometabolic risk factors linked in a much stronger way to COVID‐19 severity than to other forms of viral pneumonias may be modulating innate immune responses in a way that could be mediated by a Warburg immunological effect. Our results also suggest the need to look at therapeutic strategies for glyceamic control at earlier stages of the disease progression.

## CONFLICT OF INTEREST

The authors who have taken part in this study declared that they do not have anything to disclose regarding funding or conflict of interest related to this manuscript.

## Data Availability

The data that support the findings of this study are available from the corresponding author, upon reasonable request.
